# Municipal Solid Waste Disposal Operational Performance in Wa Municipality, Ghana

**DOI:** 10.5696/2156-9614-9.23.190903

**Published:** 2019-07-23

**Authors:** Patrick Aaniamenga Bowan, Sam Kayaga, Andrew Cotton, Julie Fisher

**Affiliations:** Water Engineering Development Centre, School of Architecture, Building and Civil Engineering, Loughborough University, Loughborough, UK

**Keywords:** municipal solid waste, operational performance, waste disposal, health impacts, scenario analysis, substance flow analysis

## Abstract

**Background.:**

The generation and management of solid waste pose potential adverse impacts on human health and the environment.

**Objective.:**

The present study examines the operational performance of municipal solid waste (MSW) disposal in the Wa Municipality, Ghana.

**Methods.:**

The study applied both qualitative and quantitative research methods and modelled the Wa Municipality's MSW disposal system using the municipal solid waste decision support tool (MSW DST). Acid gases (sulphur oxides and nitrogen oxides) and total particulate matter that have a direct impact on human health were set as the objective functions for modelling five MSW disposal scenarios. The modelled scenarios were: 1) landfill disposal only; 2) composting and landfill disposal; 3) composting, incineration, refuse derived fuels (RDF) and landfill disposal; 4) separation, composting, incineration, RDF and landfill disposal; and 5) separation, transfer, material recovery, composting, incineration, RDF and landfill disposal. The pollutants chosen as indicators for substance flow analysis included lead, cadmium, arsenic, mercury, copper, chromium, and zinc.

**Results.:**

Scenarios 4 and 5 produced the least engineering cost of 1 150 000 US $/year for the entire MSW disposal system, whereas scenario 2 produced the highest cost of 1 340 000 US $/year. Scenario 5 produced the least average health impacts of −5.812E-04 lbs/year, while scenario 2 generated the highest engineering cost and produced the highest average health impact of 9.358E-05 lbs/year. Scenarios 5 and 4, which included waste-to-energy conversion in the systems, produced the lowest average health impacts (−5.812E-04 lbs/year and −5.611E-04 lbs/year, respectively).

**Conclusions.:**

The adoption of an integrated solid waste management concept, including waste-to-energy technologies, will not only help to lessen MSW disposal hazards, but also to produce alternative sources of energy for Ghana and other developing countries.

**Competing Interests.:**

The authors declare no competing financial interests

## Introduction

A number of serious and highly publicized pollution incidents associated with improper waste management practices have led to public concern about the lack of controls, inadequate legislation, and environmental and human health impacts.^1^ A waste management hierarchy plan based on the most environmentally sound criteria favors waste prevention/minimization, waste reuse, recycling, and composting.^2^–^4^

Despite important technological advancements, including improved legislation and regulatory systems in the field of waste management and more sophisticated health surveillance, the public acceptance of locating waste disposal and treatment facilities in close proximity to human populations is still very low due to concerns about adverse health and environmental effects.^1^ Health issues are associated with every step of the handling, treatment, and disposal of waste, both directly (through recovery and recycling activities or other occupations in the waste management industry, by exposure to hazardous substances in the waste, or to emissions from incinerators and landfill sites, vermin, odors and noise), or indirectly (through ingestion of contaminated water, soil, and food).^1^,^5^,^6^

The health impacts of solid waste are varied and may depend on numerous factors including the nature of the waste, duration of exposure, population exposed, and availability of prevention and mitigation interventions.^7^ Impacts may range from mild psychological effects to severe morbidity, disability, or death. Nevertheless, the literature on the health impacts of solid waste remains limited and inconclusive and there is no clear evidence of adverse health outcomes for the general population from waste management, despite widespread concern over the health impacts of landfills.^1^,^8^,^9^,^10^ Studies on health impacts from landfills show that living near a waste site is associated with adverse health effects, ranging from allergies to cancer and birth defects.^11^ Similarly, Giusti^1^ indicates that there is convincing evidence of a high risk of gastrointestinal problems associated with pathogens originating at waste treatment plants.^1^

In general, environmental pressures from the generation and management of solid waste include emissions into the air, water and soil, and pose potential impacts on human health and the environment.^11^,^12^ Thus, environmental policies and strategic measures are required to reduce waste emission and improve waste management practices.^13^ Consequently, the foundation of modern waste management is a combination of regulation, design, construction, operation, maintenance, and monitoring features to create an inter-dependent, overlapping system to protect human health and the environment.^14^–^17^

### Baseline Scenario in Wa Municipality, Ghana

The most commonly practiced municipal solid waste (MSW) disposal option in the Wa Municipality and the whole of Ghana (as in many other developing countries) involves the collection of mixed waste materials and subsequent dumping at designated sites.

**Figure 1:**
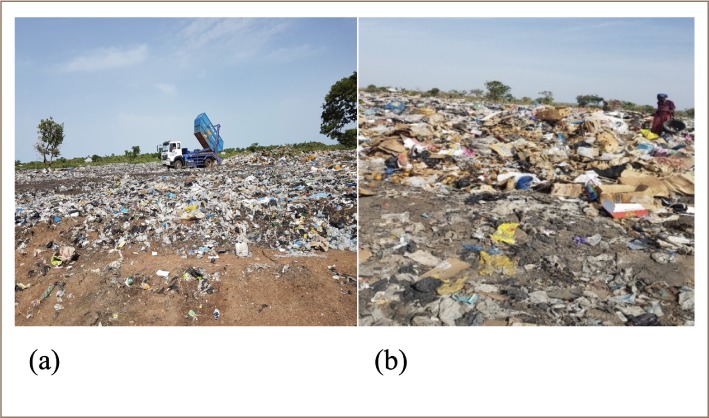
Open waste dumping at the Wa Municipality disposal site (1:100 scale)

In the Wa Municipality, all of the collected solid waste from residential and commercial areas, institutions, and streets are carried to a dumping ground *(Figure 1)* at *Siriyiri. Siriyiri* is located in a separate district - the Wa West District. The *Siriyiri* disposal site was created in 2001 and has been poorly managed, without any formal material recovery, however, some informal material recovery is undertaken by scavengers (informal waste collectors).

**Abbreviations**
*ISWM*: Integrated solid waste management
*MSW*: Municipal solid waste
*MSW DST*: Municipal solid waste decision support tool
*RDF*: Refuse-derived fuels
*TPM*: Total particulate matter
*WTE*: Waste-to-energy

The MSW flow in the Wa Municipality begins at waste generation sources (households, commercial areas, institutions, and streets). Waste segregation, the technique by which solid waste is divided into its components (organic and inorganic), is not undertaken at the generation point nor throughout the waste management chain. As a result, municipal authorities do not have a good understanding of the MSW generation or characteristics in the municipality.

Some MSW generators dispose of their waste inappropriately, by discarding into bushes, open burning, and by burying in pits. Municipal solid waste that is disposed of with these methods do not enter the MSW stream and are not managed by the municipal authorities.

**Figure 2:**
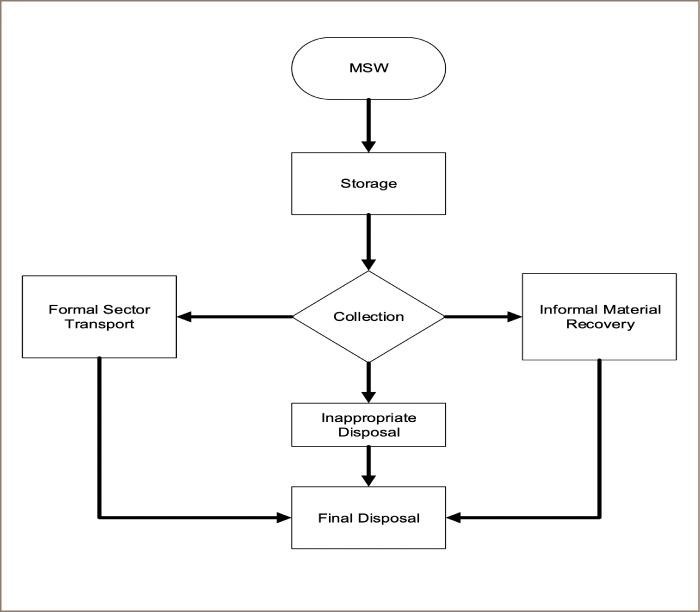
Municipal solid waste flow in the Wa Municipality

Municipal solid waste collection is undertaken by both the formal (municipal authorities and Zoomlion Ghana Limited, the only private waste collection company engaged in the Wa Municipality) and informal (waste merchants and scavengers) sectors. Informal waste collectors transport all of the collected waste to designated dumping sites, usually near the waste merchant's residence, for onward transportation to the southern part of Ghana for sale. The formal sector transports all of the mixed collected waste to the main disposal site (un-engineered open dumping site) at *Siriyiri* for final disposal. Figure 2 illustrates the MSW flow in the Wa Municipality.

**Figure 3:**
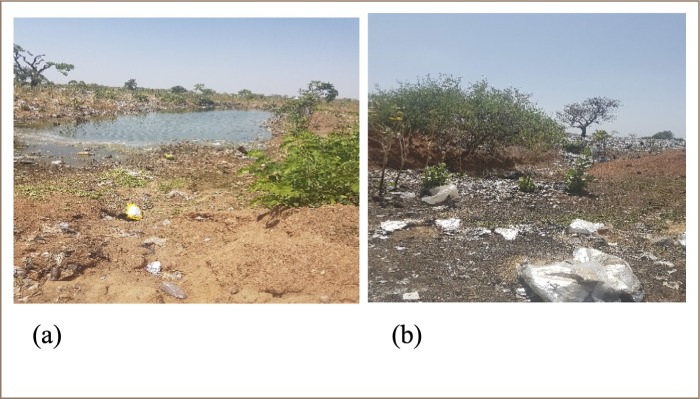
Waste disposed in low-lying areas at the Wa Municipality's disposal site (1:100 scale)

The *Siriyiri* disposal site is characterized by a low-lying area with a borehole located 300 m away from the disposal site without any precautionary measures. Both liquid (human excreta) and solid wastes are disposed of in the same dumping site *(Figure 3)*. The borehole water was not tested to determine its quality, although there is high potential for contamination by leachate from the disposal site. The manager of the disposal site reported that the *Siriyiri* community has protested the location of the disposal site on several occasions, to no avail, and that it represents a breach of environmental justice.

Municipal solid waste disposal practices in the Wa Municipality and Ghana in general consist of waste collection, transportation, and open dumping, and the majority of waste is openly dumped without pre-treatment.

Thus, sustainable waste management has remained elusive in the Wa Municipality and in Ghana as a whole.

The present study examines the operational performance of municipal solid waste (MSW) disposal in the Wa Municipality, Ghana, with a focus on health impacts of MSW disposal through scenario modelling of five MSW disposal scenarios, using the municipal solid waste decision support tool (MSW DST).^18^

The MSW DST was adopted for this study as it can evaluate various MSW management options and optimizes their environmental burdens, is applicable to both small and large waste management systems, and the developers of the tool allowed it to be used free of charge for the present study.

The MSW DST is the outcome of a cooperative research agreement with the Research Triangle Institute International (co-funded by the Environmental Protection Agency and United States Department of Energy) which started in the mid-90s. The Research Triangle Institute led a team comprised of academic institutions and research firms through the complex task of building this tool, enabling users to compare the results of different SW management scenarios.

## Methods

The evaluation of MSW disposal operational performance in the Wa Municipality, Ghana was based on the formulation, construction, optimization and scenario analysis of five modelled MSW disposal options through the combination of material flow analysis and substance flow analysis. The data was obtained from both primary and secondary sources, using qualitative and quantitative research methods. The primary data was obtained through passive observation of MSW disposal activities in the Wa Municipality, and the secondary data was obtained through reviewing official reports and journal publications.

Five (5) MSW disposal scenarios, reflecting different MSW disposal systems, were modelled and compared using the MSW DST based on their ability to improve the current situation of MSW disposal in the Wa Municipality. Since the scenarios were assumed not to influence MSW generation, the same amounts and composition of MSW were used in all 5 scenarios. Additionally, the acid gases (sulphur oxides (SOx), nitrogen oxides (NOx)) and total particulate matter (TPM) that have a direct impact on human health were chosen as the objective functions for optimization in the five scenarios.

Nitrogen oxides plays a major role in several environmental and health effects. Breathing air with a high concentration of NOx can irritate airways in the human respiratory system, and exposures of even short duration can aggravate respiratory diseases, particularly asthma, leading to respiratory symptoms (such as coughing, wheezing or difficulty in breathing).^19^

Similarly, exposure to SO_X_ in ambient air has been associated with reduced lung function, increased incidence of respiratory symptoms and diseases, irritation of the eyes, nose, and throat, and premature mortality.^20^

Particulate matter also poses a threat to human health. Tiny particles usually less than 10 micrometers in diameter pose a risk, as they can easily enter human lungs, and possibly enter the bloodstream.^21^

For the substance flow analysis, lead, cadmium, arsenic, mercury, copper, chromium, and zinc were chosen as indicators (pollutants) for all five scenarios. The health impacts of these pollutants, assessed through the objective functions of the modelling (NOx and SO_X_ and TPM), were categorized as cancer air, cancer water, non-cancer air, and non-cancer water health impacts.

### Conceptual model formulation of the scenario analysis

The MSW disposal system modelled was the Wa Municipality's MSW disposal system. The processes that were modelled included waste generation, collection, transfer, separation (material recovery), composting, combustion, refuse-derived fuels (RDF), and disposal in a landfill. Five MSW disposal scenarios were formulated, built and analyzed based on uncertainty and sensitivity analysis with the objective of minimizing environmental burdens.

The optimization module of the MSW DST is implemented using CPLEX linear programming solver and is constrained by mass flow equations based on the quantity and composition of waste entering each unit process in the waste management system (i.e., collection, recycling, treatment, and disposal options).

The optimization module uses linear programming techniques to determine the optimum solution consistent with the specified objective, constraints, and mass flow.

The MSW DST modelling process consists of four basic components: process models, waste flow model, optimization model, and a graphic user interface. The process models consist of a set of spreadsheets developed in Microsoft Excel. These spreadsheets use a combination of default and user-supplied data to calculate the cost and life cycle inventory, with the coefficients on a per unit mass basis for the MSW components being modelled for each SW management unit process (collection, transfer, treatment, and disposal). There are a total of eight steps, but five steps are required to complete modelling a scenario.

These steps are presented in Table 1.

**Table 1:** Steps in the Municipal Solid Waste Decision Support Tool

	**Step**	**Description**
1.	Define generation^*^	Define generation sectors to include in the model scenario analysis. The parameters for residential sectors include the population, generation rate (kg/person/day), household population density (people/house), and the parameter for commercial sectors (the number of commercial units and generation rate).
2.	Select processes^*^	Select processes to include in the model and scenario analysis (waste collection, transfer, material recovery facility, treatment, and landfill disposal methods).
3.	Select report options^*^	Select objective function.
4.	Specify process input	Input site-specific information for the process.
5.	Build model^*^	Creates the life cycle inventory.
6.	Set process constraints	Specify constraints (if any).
7.	Set diversion targets	Define which processes can divert waste (recycling and composting) and the target of diversion in percentages.
8.	Solve and view report^*^	Three - four reports can be created: impact assessment, cost and inventory analysis, recycling, and mass flow reports.
Steps used to solve the problem in the present study	The daily waste generation of Wa Municipality, household MSW composition and chemical properties in Ghana were considered as the input of the residential sector, as illustrated in Table 2 and 3, respectively. The Wa market waste average daily generation of 0.23 kg/day and MSW composition, shown in Table 4, was also considered as the input of the commercial sector. These were used for modelling five scenarios that represented different degrees of separation at the source and different treatment configurations to obtain optimal solutions for each scenario.	

^*^steps required to complete modelling a scenario

The optimization module uses linear programming techniques to determine the optimum solution consistent with the specified objective, mass flow, and specified constraints. Thus, the main objective function of the modelling and optimization in this study was to minimize the health impacts of MSW disposal.

The categories of MSW environmental impacts include human health, greenhouse effect (global warming), acidification, eutrophication, and photochemical ozone synthesis. However, this study was limited to only the human health impact category of MSW and aimed to optimize the minimization of the environmental burdens of acid gases (NOx and SO_X_) and particulate matter that have direct impacts on human health.

Additionally, the following (seven) substances were chosen as indicators for the substance flow analysis: lead, cadmium, arsenic, mercury, copper, chromium, and zinc.

Lead, copper, zinc, arsenic, and chromium in landfills and leachates determines the long-term rehabilitation of the environment.^22^,^23^ These compounds affect air, surface and groundwater qualities, as well as pose a threat to human health, as some can cause mild mental retardation and cardiovascular diseases.^24^–^27^ Cadmium, mercury, and lead are also indicators for the presence of toxic metals in the atmosphere.^23^ Five scenarios were conducted to determine the optimal MSW disposal system based on low engineering costs and minimal environmental burdens. The aim of the modelling and optimization using the MSW DST is to increase decision-makers' awareness with the results of this research in order to reduce the undesirable environmental effects of MSW disposal in the future. Therefore, the results were analyzed on an inventory of stressors by the health impact category of the modelled scenarios.

### Functional unit

The functional unit was chosen as the average amount of municipal generated waste in the Wa Municipality per day in the residential sectors based on the residential typology/income level (compound-house/low-income, semi-detached/middle-income, and single-unit/high-income residential dwellings) and one commercial generation sector (the Wa market).

The daily waste generation of Wa (average daily generation of 0.25 kg/capita/day and 32 ton/day based on the 2017 population projection of 128 873)^28^ and household MSW composition and chemical properties in Ghana were considered as the input of the residential sector, as illustrated in Table 2 and 3, respectively. The commercial sector input included the Wa market average daily waste generation of 0.23 kg/day and MSW composition, as shown in Table 4. Thus, the modelled systems consisted of inputs from the residential and commercial sectors.

**Table 2:** Household Waste Composition and Generation in Ghana (adapted from Miezah et al.^28^)

**Component**	High income areas (%) ^*^	Middle income areas (%) ^*^	Low income areas (%) ^*^	**Average (%)**
Yard waste (leaves)	17.334	7.562	8.915	11.270
Animal dropping/manure (grass)	0.176	0.379	0.291	0.282
Wood (branches)	1.301	1.346	1.282	1.310
News paper	0.674	0.388	0.414	0.492
Cardboard	3.223	3.215	2.233	2.890
Office paper	0.605	0.445	0.541	0.530
Tissue paper	1.148	1.520	1.677	1.448
HDPE - Translucent	3.075	2.751	3.418	3.081
HDPE - Pigmented	2.071	3.628	5.358	3.686
PET	3.315	3.297	2.104	2.905
PP rigid	1.554	1.521	1.126	1.400
PS	0.606	0.538	0.583	0.576
PVC	0.554	0.618	0.247	0.473
Other plastics	2.402	1.983	2.153	2.179
Ferrous can	1.721	1.319	2.108	1.716
Ferrous metals	1.060	1.575	0.530	1.055
Plain glass	0.846	1.072	0.588	0.835
Colored glass	2.864	1.991	0.00	1.618
Leather and rubber	1.012	1.171	1.035	1.073
Food waste	44.201	50.595	49.358	48.051
Textiles	0.528	1.149	1.799	1.159
Miscellaneous	9.73	11.937	14.24	11.969
Total	100	100	100	100

Abbreviations: HDPE, high-density polyethylene; PET, polyethylene terephthalate; PP, polypropylene; PS, polystyrene; PVC, polyvinyl chloride.

^*^High income area: per capita daily consumption above $20 (the houses are often detached single buildings with large compound either paved or grassed) Middle income area: per capita daily consumption of between $4 and $20 (residential areas are characterized by flats or bungalows and often occupied by more than one household) Low income area: per capita daily consumption below $4 (areas with poor social services and amenities)

**Table 3:** Chemical Composition of Household Wastes in Ghana

**Property**	**Composition according to Kuleape *et al.*^a^**	**Composition according to Fobil *et al.*^b^**	**Composition according to Adu *et al.*^c^**
Calorific value (kJ/kg)	1.39 × 10^4^ – 2.99 × 10^4^	1.4 × 10^4^ – 2.0 × 10^4^	1.69 × 10^4^
Moisture content (%)	25 - 76	40 - 60	50
Ash content (%)	2.2 - 19	nd	nd
Volatile solids (%)	31 - 88	nd	nd
Density (kg/m^3^)	nd	5.3 × 10^2^ – 5.4 × 10^2^	nd

Abbreviation: nd, not determined.

^a^ Adapted from Kuleape *et al.^29^*

^b^ Adapted from Fobil *et al. 30*

^c^ Adapted from Adu *et al. 31*

**Table 4:** Wa Market (Commercial) Waste Consumption ^a^

**Composition**	**Percentage (%)**
Cardboard	13.1
Organics (combustible compostable recyclable)	46.6
PET	4.9
Textiles (combustible non-compostable recyclable)	3.4
Ferrous cans)	2.6
Other (non-combustible non-compostable non-recyclable)	29.4
Total	100

Abbreviation: PET, polyethylene terephthalate.

^a^ Adapted from Bowan *et al.*^32^

### Limitations of the scenario analysis

The researchers acknowledge key assumptions and limitations of the present analysis. Studies to characterize the quantity and composition of MSW are often cited as a key factor in selecting waste management processes.^33^,^34^ The present study applied Ghana and Wa Municipality waste characterization data available in the literature in the modelling and analysis, but could not determine the data quality. The modelling relied on some default data in the model because of the non-availability of some site-specific data from Ghana and Wa Municipality. The MSW DST does not include models for all possible waste disposal technologies. Therefore, anaerobic digestion and new or emerging technologies, such as waste gasification and pyrolysis were not considered. The study did not place a limit on the amount of waste that any process can accept. In practice, facilities are designed to handle a certain minimum or maximum capacity of waste and, therefore, would be limited in the amount of waste they could process.

## Results

The present study identified a number of shortcomings in the Wa Municipality disposal system. Most of the population is unconnected to the waste collection system. There is no waste separation at the source and no formal material recovery/recycling from waste. Municipal authorities have no knowledge of waste generation rates and characteristics. Landfilling practices do not comply with the best available technology for sanitary landfilling and landfills are an ineffective use of space. Additionally, open dumping of biodegradable waste results in long-term emissions (gas and leachate). Lastly, there is no integrated stormwater management in place.

### Scenario 1 — Landfill disposal only

Sanitary landfilling is the recommended MSW disposal option for most developing countries and is the desired disposal system in Ghana. For this scenario, all mixed MSW was collected and disposed of in a sanitary landfill and the human impact categories evaluated to determine the environmental impacts.

**Figure 4:**
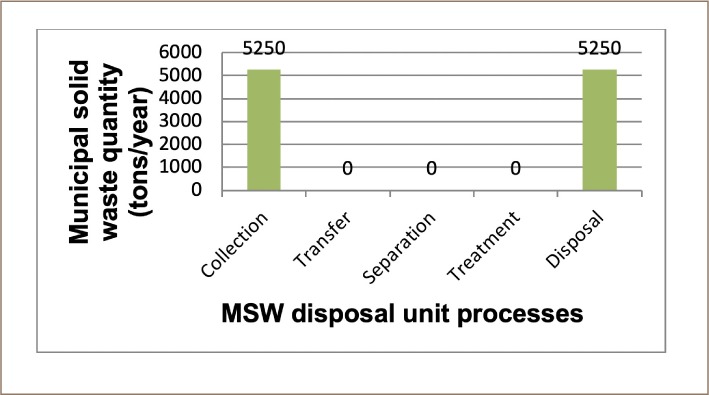
Municipal solid waste mass flow in scenario 1

The optimal solutions found for NO_X_, SO_X_, and TPM as the optimizing objectives for scenario 1 were 5970, 1890, and 358 lbs/year, respectively, and the engineering cost for the entire system was 1 210 000 US $/year. There was no change in the mass flow for all three optimizing objectives, as a total mass flow of 5250 metric tons/year was disposed of in the landfill. Figure 4 shows the mass flow of waste for scenario 1.

The values of the chosen pollutants (lead, cadmium, arsenic, mercury, copper, chromium, and zinc) and their impact categories are presented in Table 5. The biggest pollutant for all three optimizing objectives in this scenario was cadmium (9.38E-08 lbs/year) under the cancer water impact category, followed by lead (8.4E-05 lbs/year) under the non-cancer air category for both NO_X_ and SO_X_ as optimizing objectives, and 9.4E-05 lbs/year under the non-cancer air category for optimizing objective TPM. Under the non-cancer water category, copper was the least pollutant (1.28E-09 lbs/year) for all three optimizing objectives.

**Table 5:** Inventory of Human Health Impact Categories for Scenario 1

		**Objective function**		

**Impact categories**	**Pollutant name**	**Nitrogen oxides (5970 lbs/yr)**	**Sulphur oxides (1890 lbs/yr)**	**Total particulate matter (358 lbs/yr)**
Cancer air	Lead	**Value (lbs/year)**		

		2.39E-07	2.39E-07	2.39E-07

Cancer water	Cadmium	9.38E-08	9.12E-08	9.12E-08
	Arsenic	2.38E-05	2.38E-05	2.38E-05
	Mercury	3.33E-07	3.33E-07	3.33E-07
	Lead	4.62E-08	4.62E-08	4.62E-08

Non-cancer air	Lead	8.40E-05	8.40E-05	9.40E-05

Non-cancer water	Copper	1.28E-09	1.28E-09	1.28E-09
	Cadmium	2.45E-05	2.45E-05	2.45E-05
	Arsenic	1.77E-03	1.77E-03	1.77E-03
	Mercury	3.94E-05	3.94E-05	3.94E-05
	Chromium	1.56E-09	1.56E-09	1.56E-09
	Lead	1.62E-05	1.62E-05	1.62E-05
	Zinc	1.57E-05	1.57E-05	1.57E-05

**Average**		**1.519E-04**	**1.519E-04**	**1.519E-04**

**Average of the three objective functions**			**1.519E-04**	

### Scenario 2 — Composting and landfill disposal

**Figure 5:**
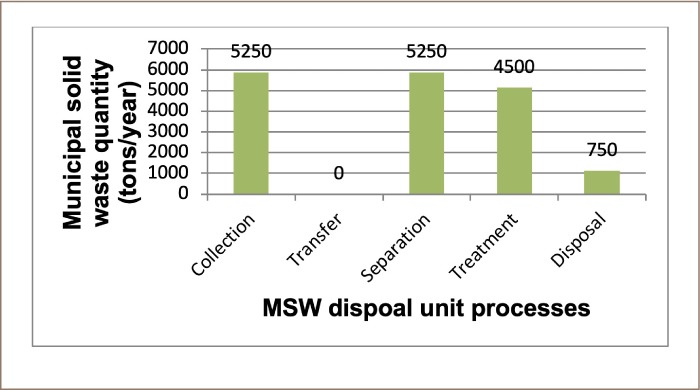
Municipal solid waste mass flow in scenario 2

Composting and sanitary landfilling are the two most commonly recommended waste management options for the organic waste fraction, especially in developing countries. For scenario 2, all the collected mixed MSW (5250 tons/year) was first sent to a separation plant and the mixed waste sorted into organic and inorganic components. The organic component of 4500 metric tons/year was processed through composting, 386 tons/year of inorganic MSW and 436 tons/year of non-compostable organic MSW were disposed of in a landfill. The mass flow of the waste is presented in Figure 5.

The optimal solutions for NO_X_, SO_X_, and TPM as the optimizing objectives were 85.7, −3490, and −2630 lbs/year, respectively. The total engineering cost for scenario 2 was 1 340 000 US $/year. The pollutant (lead, cadmium, arsenic, mercury, copper, chromium, and zinc) values and their impact categories are presented in Table 6. Lead under the cancer air impact category was the least pollutant (−3.83E-08 lbs/year) for NO_x_ as the optimizing objective. The optimizing objective SO_X_ under the cancer air impact category also found lead to be the least pollutant (−4.25E-08 lbs/year), whereas optimizing objective TPM showed cadmium under the non-cancer water impact category to be the least pollutant (1.02E-04 lbs/year).

**Table 6:** Inventory of Human Health Impact Categories for Scenario 2

		**Objective function**		

**Impact categories**	**Pollutant name**	**Nitrogen oxides (85.7 lbs/yr)**	**Sulphur oxides (−3490 lbs/yr)**	**Total particulate matter (−2630 lbs/yr)**
Cancer air	Lead	**Value (lbs/year)**		

		−3.83E-08	−4.25E-08	3.08E-09

Cancer water	Cadmium	3.69E-07	3.68E-07	3.78E-07
	Arsenic	4.30E-06	4.28E-06	4.44E-06
	Mercury	3.17E-08	2.60E-08	7.17E-08
	Lead	1.34E-07	1.33E-07	1.41E-07

Non-cancer air	Lead	−1.34E-05	−1.49E-05	1.08E-06

Non-cancer water	Copper	7.66E-08	7.66E-08	7.65E-08
	Cadmium	9.90E-05	9.88E-05	1.02E-04
	Arsenic	3.19E-04	3.17E-04	3.29E-04
	Mercury	3.75E-06	3.07E-06	8.49E-06
	Chromium	2.99E-10	2.67E-10	5.10E-10
	Lead	4.69E-05	4.67E-05	4.95E-05
	Zinc	7.46E-04	7.46E-04	7.47E-04

**Average**		**9.278E-05**	**9.242E-05**	**9.555E-05**

**Average of the three objective functions**			**9.358E-05**	

### Scenario 3 — Composting, combustion, refuse-derived fuels, and landfill disposal

In scenario 3, MSW was collected and transported to a sorting plant for separation and subsequently taken to various processing/treatment plants. Compostable organic MSW was sent to a composting facility, inorganic MSW was sent to combustion and RDF facilities. Non-compostable and non-combustible MSW together with the residues of the composting, combustion and RDF processes were disposed of in a landfill.

**Figure 6:**
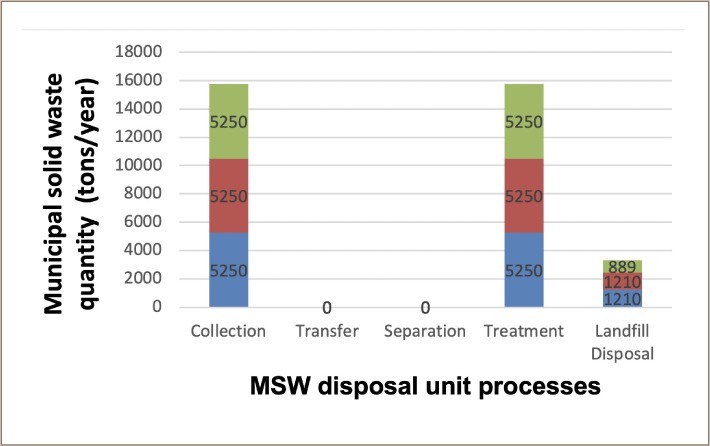
Municipal solid waste mass flow in scenario 3

For this scenario, the mass flow for NO_X_ and SO_X_ as optimizing objectives involved 5250 metric tons/year of MSW sent to the RDF facility, resulting in 1210 metric tons/year of residue (ash) disposed of in a landfill. However, for TPM as the optimizing objective, 5250 metric tons/year of MSW were sent to a mixed combustion treatment plant, which resulted in 889 metric tons/year of residue (ash) disposed of in a landfill. The mass flow of waste for scenario 3 is illustrated in Figure 6.

Scenario 3 had negative values of −5250, −45 700, and −4710 lbs/year as the optimal solutions for NO_X_, SO_X_, and TPM as optimizing objectives, respectively. The engineering cost for scenario 3 system was 1 200 000 US $/year, which is slightly lower than the engineering cost for scenario 1 by 10 000 US $/year.

The health impact categories and their pollutant values are shown in Table 7. This scenario showed that arsenic under the cancer water impact category was the least pollutant for NO_X_ and SO_X_ optimizing objectives (−9.35E-06 lbs/year), while mercury under the cancer water impact category of −9.51E-09 lbs/year was the least pollutant for TPM as the optimizing objective.

**Table 7:** Inventory of Human Health Impact Categories of Scenario 3

		**Objective function**		

**Impact categories**	**Pollutant name**	**Nitrogen oxides (−5250 lbs/yr)**	**Sulphur oxides (−45700 lbs/yr)**	**Total particulate matter (−4710 lbs/yr)**
Cancer air	Lead	**Value (lbs/year)**		

		−5.50E-06	−5.50E-06	−1.62E-06

Cancer water	Cadmium	1.56E-08	1.56E-08	1.89E-08
	Arsenic	−9.35E-06	−9.35E-06	−6.08E-06
	Mercury	−1.46E-08	−1.46E-08	−9.51E-09
	Lead	−1.57E-08	−1.57E-08	−1.02E-08

Non-cancer air	Lead	−1.93E-03	1.93E-03	−5.7E-04

Non-cancer water	Copper	−5.65E-08	−5.65E-08	−3.67E-08
	Cadmium	4.19E-06	4.19E-06	5.07E-06
	Arsenic	−6.92E-04	−6.92E-04	−4.51E-04
	Mercury	−1.73E-06	−1.73E-06	−1.13E-06
	Chromium	−4.13E-10	−4.13E-10	−2.53E-10
	Lead	−5.49E-06	−5.49E-06	−3.58E-06
	Zinc	−2.68E-04	−2.68E-04	−1.72E-04

**Average**		**−2.237E-04**	**−2.237E-04**	**−9.234E-05**

**Average of the three objective functions**			**−5.530E-04**	

### Scenario 4 — Source separation, composting, combustion, refuse-derived fuels, and landfill disposal

Scenario 4 was similar to scenario 3, except that in scenario 4, there was segregation of MSW into organic and inorganic MSW at the point of generation for collection. The organic MSW was transported to a composting plant for treatment/processing, whereas the inorganic MSW was transported to combustion and RDF facilities for treatment/processing.

There were different mass flows for all three optimizing objectives. For NO_X_ as an optimizing objective, the entire 5250 metric tons/year of MSW was first sent to a front-end mixed separation point. After the separation, 4580 metric tons/year of organic MSW was sent to a composting facility for processing/treatment, whereas 434 metric tons/year of MSW was directly disposed of in a sanitary landfill. The composting process generated 568 metric tons residue, which was disposed of in a landfill.

Similarly, with SO_X_ as the optimizing objective, 558 metric tons/year of pre-sorted recyclables were taken to a recycling plant and 4700 metric tons/year of MSW were sent to a RDF facility to produce pellets. The RDF process produced a residue of 1080 ton of ash, which was disposed of in a landfill.

**Figure 7:**
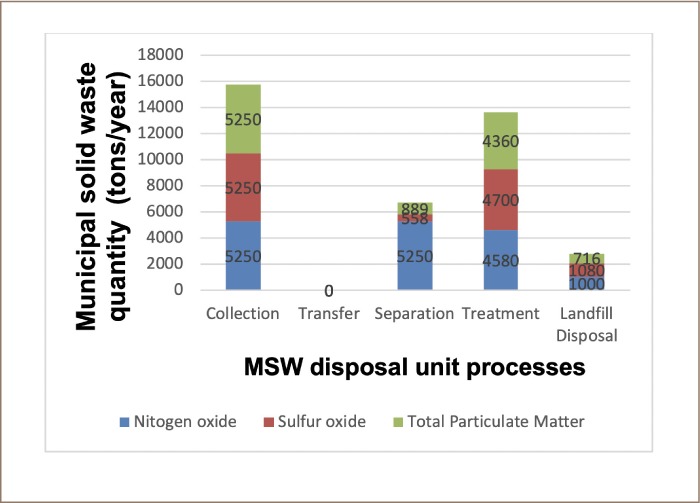
Municipal solid waste mass flow in scenario 4

For the TPM as an optimizing objective, 890 metric tons/year of recyclables were sorted from the total 5250 metric tons/year of MSW and 4360 metric tons/year of MSW was taken to a mixed combustion facility for waste-to-energy (WTE) conversion. The combustion process produced 716 metric tons/year of ashes which were disposed of in a landfill. The mass flows of the waste for scenario 4 are shown in Figure 7.

The optimal engineering cost for scenario 4 was 1 150 000 US $/year, which is lower than the engineering cost for scenarios 1, 2, and 3. Optimizing objectives SO_X_ and TPM had negative optimal solutions, −19800 and −4520 lbs/year, respectively, while objective function NO_X_ had a positive lower optimal solution of 71.7 lbs/year, which is far lower than the NO_X_ optimal solution for scenario 1 (5970 lbs/year). Table 8 presents the health impacts and their corresponding pollutant values.

**Table 8:** Inventory of Human Health Impact Categories of Scenario 4

		**Objective function**		

**Impact categories**	**Pollutant name**	**Nitrogen oxides (71.7 lbs/yr)**	**Sulphur oxides (−19800 lbs/yr)**	**Total particulate matter (−4520 lbs/yr)**
Cancer air	Lead	**Value (lbs/year)**		

		−2.75E-08	−1.59E-06	3.08E-07
Cancer water	Cadmium	3.3E-07	−6.8E-08	1.36E-08
	Arsenic	3.85E-06	−1.78E-04	−7.42E-05
	Mercury	2.83E-08	−1.31E-07	−5.44E-08
	Lead	1.2E-07	−2.95E-07	−1.23E-07

Non-cancer air	Lead	−9.64E-06	−5.56E-04	1.08E-04

Non-cancer water	Copper	6.84E-08	−3.62E-07	−1.5E-07
	Cadmium	8.85E-05	−1.83E-05	3.66E-06
	Arsenic	2.85E-04	−1.31E-02	−5.5E-03
	Mercury	3.35E-06	−1.55E-05	−6.44E-06
	Chromium	2.67E-10	−8.51E-09	−3.49E-09
	Lead	4.2E-05	−1.03E-04	−4.32E-05
	Zinc	6.66E-04	−2.63E-03	−8.48E-04

**Average**		**8.304E-05**	**−1.277E-03**	**−4.892E-04**

**Average of the three objective functions**			**−5.611E-04**	

This scenario produced varied pollutants values for all three optimizing objectives. Optimizing objective NO_X_ had the least pollutant for lead (−9.64E-06 lbs/year) under the non-cancer air impact category and the highest pollutant for cadmium (8.85E-05 lbs/year) under the non-cancer water impact category. Similarly, SO_X_ as the optimizing objective showed chromium (−8.51E 09 lbs/year) and lead (−1.03E-04 lbs/year) to be the least and highest pollutants under the non-cancer water impact category, respectively. For TPM as the optimizing objective, zinc (−8.48E-04 lbs/year) under the non-cancer water was the least pollutant and cadmium (3.66E-06 lbs/year) was the highest pollutant under the non-cancer water category.

### Scenario 5 — Source separation, transfer stations, material recovery facility, composting, combustion, refuse-derived fuels, and landfill disposal

In scenario 5, MSW is separated at the source, transported to transfer stations, and subsequently transferred to a material recovery facility before finally being sent for treatment/processing in composting, combustion, and RDF facilities. Some MSW and residue of the processing were disposed of in a sanitary landfill. Like scenario 3, in scenario 5 all three optimizing objectives have negative optimal solutions: −3820, −19 900, and −4520 lbs/year for NO_X_, SO_X_, and TPM, respectively.

**Figure 8:**
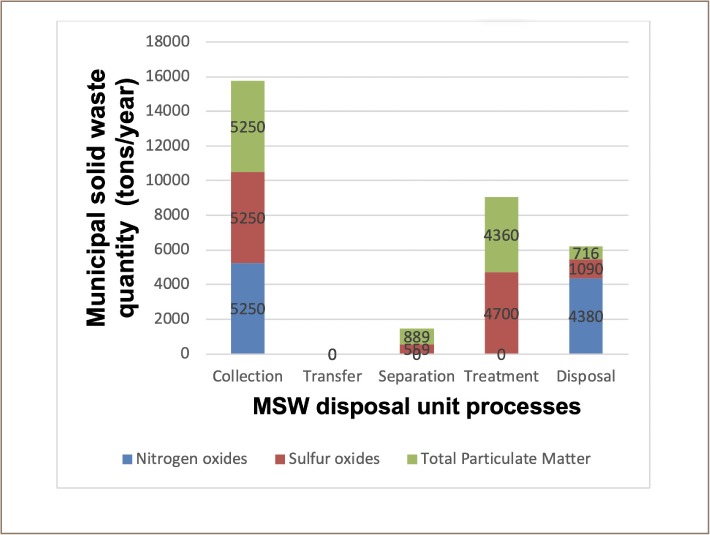
Municipal solid waste mass flow in scenario 5

Scenario 5 equally produced different mass flows for the three optimizing objectives. The mass flows of scenario 5 are shown in Figure 8. Optimizing objective NO_X_ involved 43870 metric tons/year of MSW of the total 5250 metric tons/year disposed of in a landfill with the possibility of methane capture. Sulphur oxides involved 559 metric tons/year of comingled recyclables taken out of the 5250 metric tons/year of MSW for recycling, and 4700 metric tons/year of mixed MSW was sent for WTE conversion in a combustion facility. The WTE conversion resulted in 1090 metric tons/year of ashes, which were disposed of in a landfill. For TPM as the optimizing objective, 889 metric tons/year of recyclables were recovered for recycling and 4360 metric tons/year of MSW were sent for WTE conversion in a combustion facility. The combustion produces 716 metric tons/year of ashes which were disposed of in a landfill.

The engineering optimal cost for scenario 5 was 1 150 000 US $/year, which is the same as the cost for scenario 4 disposal system. The human health impact categories and their pollutants values are presented in Table 9. Nitrogen oxides optimizing objective showed lead to be the least pollutant in the cancer air impact category at −7.13E-07 lbs/year and in the non-cancer water impact category, mercury was shown to be the highest pollutant at 9.35E-06. Optimizing objective SO_X_ showed chromium to be the least pollutant in the non-cancer water impact category at −8.52E-09 lbs/year and lead as the highest pollutant in the non-cancer water impact category at −1.03E-04 lbs/year. For TPM as an optimizing objective, zinc (−8.48E-04 lbs/year) was the least pollutant and cadmium (3.66E-06 lbs/year) was the highest, both under the non-cancer water impact category.

**Table 9:** Inventory of Human Health Impact Categories in Scenario 5

		**Objective function**		

**Impact categories**	**Pollutant name**	**Nitrogen oxides (−3820 lbs/yr)**	**Sulphur oxides (−19900 lbs/yr)**	**Total particulate matter (−4520 lbs/yr)**
Cancer air	Lead	**Value (lbs/year)**		

		−7.13E-07	−1.58E-06	3.08E-07

Cancer water	Cadmium	4.75E-08	−7.54E-08	1.36E-08
	Arsenic	1.23E-06	−1.77E-04	−7.42E-05
	Mercury	7.9E-08	−1.31E-07	−5.44E-08
	Lead	9.28E-09	−2.95E-07	−1.23E-07

Non-cancer air	Lead	−2.5E-04	−5.53E-07	1.08E-04

Non-cancer water	Copper	4.57E-09	−3.61E-07	−1.5E-07
	Cadmium	1.28E-05	−2.03E-05	3.66E-06
	Arsenic	9.09E-05	−1.31E-02	−5.5E-03
	Mercury	9.35E-06	−1.55E-05	−6.44E-06
	Chromium	3.4E-10	−8.52E-09	−3.53E-09
	Lead	3.25E-04	−1.03E-04	−4.32E-05
	Zinc	4.25E-04	−2.63E-03	−8.48E-04

**Average**		**2.246E-05**	**−1.277E-03**	**−4.892E-04**

**Average of the three objective functions**			**−5.812E-04**	

## Discussion

**Figure 9:**
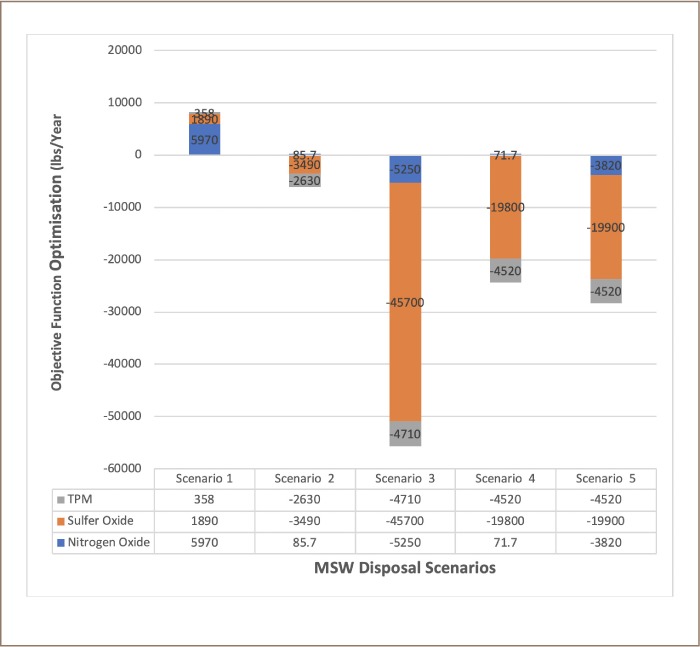
Objective functions optimizations across the five scenarios

The results showed that MSW disposal into a sanitary landfill alone does not optimize the minimization of health impacts (NO_X_, SO_X_ and TPM) compared to MSW disposal in an integrated solid waste management (ISWM) system as shown in Figure 9. This is because local environmental pollution is common in landfills due to the decomposition of waste into constituent chemicals.^35^,^36^ Meanwhile, sanitary landfilling is the most common means of MSW disposal globally and is the most cost-effective system of solid waste disposal, especially in developing countries.^37^–^39^ However, the problems of leachate and gas (methane) emissions are difficult to mitigate during the operation and decommissioning stages of landfills.^1^,^40^

**Figure 10:**
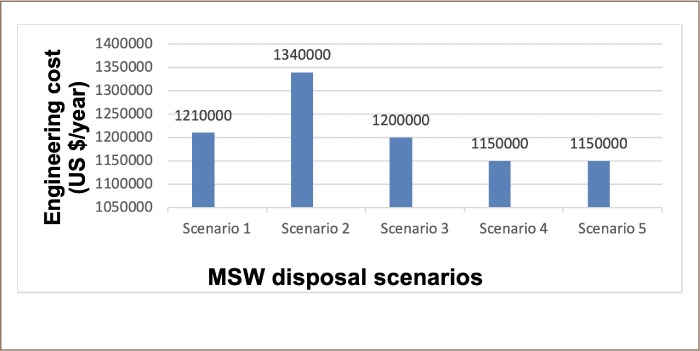
Engineering costs across the five scenarios

In terms of the engineering cost, scenarios 4 and 5 produced the lowest engineering cost of 1,150,000 US $/year for the entire MSW disposal system, whereas scenario 2 produced the highest cost of 1,340,000 US $/year, as indicated in Figure 10.

**Figure 11:**
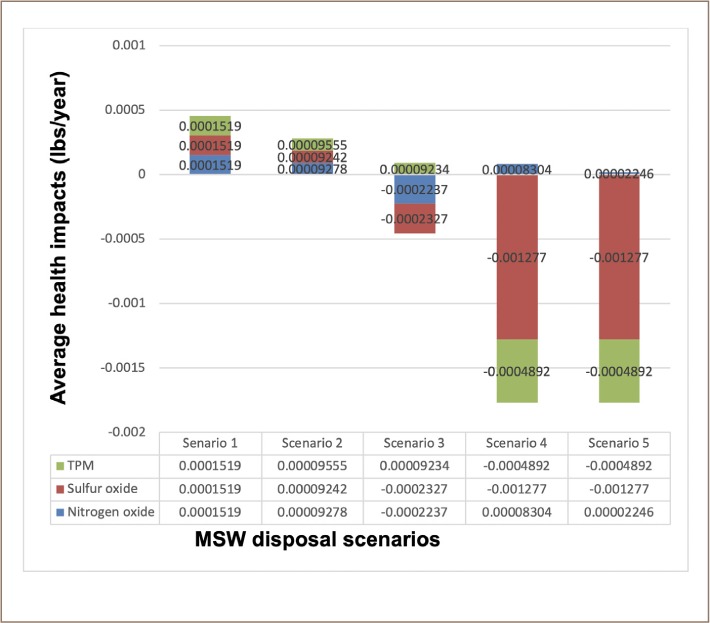
Comparison of health impacts across the five scenarios

In terms of health effects, scenario 5 produced the least average health impacts of −5.812E-04 lbs/year, while scenario 2 generated the highest engineering costs and produced the highest average health impact of 9.358E-05 lbs/year, as illustrated in Figure 11. Scenarios 4 and 5, which included WTE conversion in an ISWM system format, produced the lowest average health impacts (−5.611E-04 lbs/year and 5.812E-04 lbs/year respectively) and the lowest engineering costs.

However, WTE technologies have a poor historical image in most countries, as many countries have depended on landfills for many years, and due to the fact that many of the earlier WTE technologies such as incineration were disposal-only plants, which simply burned waste to reduce its volume.^41^,^42^ Additionally, WTE technologies tend to be among the most expensive SW management options, and require highly skilled personnel and careful maintenance.^43^,^44^ Thus, the waste management systems of most developing countries such as Ghana, which are contending with the difficulties of socio-political, technological, regulatory, financial, and human resources constraints, may not be able to effectively implement WTE technologies in an ISWM system.

Nevertheless, WTE technologies have been practiced in many developed countries, such as Japan, for decades in an effort to promote sustainable development initiatives.^41^,^45^ Waste-to-energy technologies such as incineration not only reduce the quantities of MSW, but can provide alternative sources of energy. Therefore, the implementation of WTE technologies (on small or large-scales) in developing countries such as Ghana is inevitable in the future, as WTE technologies can contribute to the reduction of the current high-power deficit affecting economic development in many developing countries.

Many researchers observe that composting (a component of scenario 2) is the cornerstone of sustainable development in the waste sector, and suggest that composting should be a widespread practice in developing countries, because it can be implemented in small and large scales.^46^–^49^However, large and centralized composting plants are often not economical, due to high operational, maintenance, and transportation costs in developing countries.^44^ The viability of commercial composting is usually dependent on the availability of a ready market for the final composted product. Subsistence farming is still widely practiced in most developing countries, with farmers depending on their own animals' droppings for manure. The demand for compost may not be able to meet the production costs in most developing countries.

## Conclusions

The present study demonstrated that the ISWM concept has the potential for optimizing the minimization of both the engineering costs and health impacts of MSW disposal. Accordingly, SW management systems that operate successfully in various parts of the world indicate that a single waste management option is not suitable to efficiently handle the full array of MSW. Thus, Ghana and other developing countries, which are overwhelmed with waste and do not have a consistent power supply for both domestic and industrial purposes, need to adopt the ISWM concept, including WTE technologies. Implementation of these technologies would help to solve the MSW disposal situation and produce alternative energy sources. Adoption of the ISWM concept in Ghana should begin with conversion of the numerous open dumping sites into sanitary landfills. This can be achieved by partitioning the existing disposal sites, such that open dumping can continue near the area where sanitary landfill cell development can begin.

## References

[1] Giusti L (2009). A review of waste management practices and their impact on human health. Waste Manag [Internet].

[2] Fudala-Ksiazek S, Pierpaoli M, Kulbat E, Luczkiewicz A (2016). A modern solid waste management strategy - the generation of new by-products. Waste Manag [Internet].

[3] Eriksson M, Strid I, Hansson PA (2015). Carbon footprint of food waste management options in the waste hierarchy – a Swedish case study. J Clean Prod [Internet].

[4] Ali SM, Pervaiz A, Afzal B, Hamid N, Yasmin A (2014). Open dumping of municipal solid waste and its hazardous impacts on soil and vegetation diversity at waste dumping sites of Islamabad city. J King Saud Univ Sci [Internet].

[5] Daley K, Castleden H, Jamieson R, Furgal C, Ell L (2015). Water systems, sanitation, and public health risks in remote communities: Inuit resident perspectives from the Canadian Arctic. Soc Sci Med [Internet].

[6] Edmunds KL, Elrahman SA, Bell DJ, Brainard J, Dervisevic S, Fedha TP, Few R, Howard G, Lake I, Maes P, Matofari J, Minnigh H, Mohamedani AA, Montgomery M, Morter S, Muchiri E, Mudau LS, Mutua BM, Ndambuki JM, Pond K, Sobsey MD, van der Es M, Zeitoun M, Hunter PR (2016). Recommendations for dealing with waste contaminated with Ebola virus: a Hazard Analysis of Critical Control Points approach. Bull World Health Organ.

[7] Ziraba AK, Haregu TN, Mberu B (2016). A review and framework for understanding the potential impact of poor solid waste management on health in developing countries. Arch Public Health.

[8] Haregu T, Ziraba A, Mberu B (2016). Integration of solid waste management policies in Kenya: analysis of coherence, gaps and overlaps. Afr Popul Stud [Internet].

[9] Olapiriyakul S (2017). Designing a sustainable municipal solid waste management system in Pathum Thani, Thailand. Int J Environ Technol Manag [Internet].

[10] Vrijheid M (2000). Health effects of residence near hazardous waste landfill sites: a review of epidemiologic literature. Environ Health Perspect.

[11] Misra V, Pandey SD (2005). Hazardous waste, impact on health and environment for development of better waste management strategies in future in India. Environ Int [Internet].

[12] Babayemi J, Ogundiran M, Osibanjo O (2016). Overview of environmental hazards and health effects of pollution in developing countries: a case study of Nigeria. Environ Quality Manag [Internet].

[13] Moh YC, Manaf LA (2014). Overview of household solid waste recycling policy status and challenges in Malaysia. Resour Conserv Recycl [Internet].

[14] Song Q, Li J, Zeng X (2015). Minimizing the increasing solid waste through zero waste strategy. J Clean Prod [Internet].

[15] Abiti B, Hartard S, Bradl H, Pishva D, Ahiakpa JK (2017). Resource prospects of municipal solid wastes generatedin the Ga East Municipal Assembly of Ghana. J Health Pollut [Internet].

[16] Barr S, Richardson D, Castree N, Goodchild MF, Kobayashi A, Liu W, Marston RA (2017). Environment and waste. International encyclopedia of geography: people, the earth, environment and technology.

[17] Rodic L, Wilson DC (2017). Resolving governance issues to achieve priority sustainable development goals related to solid waste management in developing countries. Sustain [Internet].

[18] Thorneloe SA, Weitz K, Jambeck J (2007). Application of the US decision support tool for materials and waste management. Waste Manag [Internet].

[19] Nitrogen dioxide (NO2) pollution: basic information about NO2 [Internet].

[20] (1998). Pollution prevention and abatement handbook: toward cleaner production [Internet].

[21] Particulate matter (PM) pollution: health and environmental effects of particulate matter (PM) [Internet].

[22] Hjelmar O (1996). Disposal strategies for municipal solid waste incineration residues. J Hazard Mater [Internet].

[23] Stanisavljevic N, Brunner PH (2014). Combination of material flow analysis and substance flow analysis: A powerful approach for decision support in waste management. Waste Manag Res [Internet].

[24] Suddick EC, Whitney P, Townsend AR, Davidson EA (2013). The role of nitrogen in climate change and the impacts of nitrogen–climate interactions in the United States: foreword to thematic issue. Biogeochem [Internet].

[25] Kubin E (1998). Leaching of nitrate nitrogen into the groundwater after clear felling and site preparation. Boreal Environ Res.

[26] Shonkoff SB, Hays J, Finkel ML (2014). Environmental public health dimensions of shale and tight gas development. Environ Health Perspect [Internet].

[27] Pruss-Ustun A, Corvalan C (2006). Preventing disease through healthy environments: towards an estimate of the environmental burden of disease.

[28] Miezah K, Obiri-Danso K, Kadar Z, Fei-Baffoe B, Mensah MY (2015). Municipal solid waste characterization and quantification as a measure towards effective waste management in Ghana. Waste Manag [Internet].

[29] Kuleape R, Cobbina SJ, Dampare SB, Duwiejuah SB, Amoako AB, Asare W (2014). Assessment of the energy recovery potentials of solid waste generated in Akosombo, Ghana. Afr J Environ Sci Technol.

[30] Fobil JN, Carboo D, Armah NA (2005). Evaluation of municipal solid wastes (MSW) for utilisation in energy production in developing countries. Int J Environ Technol Manag.

[31] Adu R, Lohmueller R (2012). The use of organic waste as an eco-efficient energy source in Ghana. J Environ Protect.

[32] Bowan P, Tierobaar MT (2014). Characteristics and management of solid waste in Ghanaian markets - a study of Wa Municipality. Civ Environ Res.

[33] Burnley SJ (2007). The use of chemical composition data in waste management planning - a case study. Waste Manag [Internet].

[34] Pandey BK, Vyas S, Pandey M, Gaur A (2016). Municipal solid waste to energy conversion methodology as physical, thermal, and biological methods. Curr Sci Perspect.

[35] Domingo JL, Nadal M (2009). Domestic waste composting facilities: a review of human health risks. Environ Int [Internet].

[36] Keith-Roach M, Grundfelt B, Kousa A, Pohjolainen E, Magistrati P, Aggelatou V, Olivieri N, Ferrari A (2015). Past experience of environmental, health and safety issues in REE mining and processing industries and an evaluation of related EU and international standards and regulations [Internet].

[37] Cointreau S (2004). Sanitary landfill design and siting criteria.

[38] Agamuthu P (2013). Landfilling in developing countries. Waste Manag Research [Internet].

[39] Tozlu A, Ozahi E, Abusoglu A (2016). Waste to energy technologies for municipal solid waste management in Gaziantep. Renew Sustain Energy Rev [Internet].

[40] Datta M, Kumar A (2017). Assessment of subsurface contamination potential of municipal solid waste (MSW) dumps. Indian Geotech J [Internet].

[41] (2014). Energy from waste: a guide to the debate [Internet].

[42] Arushanyan Y, Bjorklund A, Eriksson O, Finnveden G, Soderman ML, Sundqvist JO, Stenmarck A (2017). Environmental assessment of possible future waste management scenarios. Energ [Internet].

[43] Rand T, Haukohl J, Marxen U (2000). Municipal solid waste incineration: a decision maker's guide [Internet].

[44] Mudhoo A, Somaroo GD, Mohee R, Mohee R, Simelane T (2015). Treatment of solid waste: principles, key technologies and selected case studies from Africa. Future directions of municipal solid waste management in Africa.

[45] Kadir SA, Yin CY, Sulaiman MR, Chen X, El-Harbawi M (2013). Incineration of municipal solid waste in Malaysia: salient issues, policies and waste-to-energy initiatives. Renew Sustain Energy Rev [Internet].

[46] Lehmann J, Joseph S (2015). Biochar for environmental management: science, technology and implementation.

[47] Salim MR, Noor ZZ, Tin LC, Okabe K, Shaharudin N, Othman SN, Ting TS, Tsong TB (2014). Sustainable waste management towards a low carbon society.

[48] Kane D (2015). Carbon sequestration potential on agricultural lands: a review of current science and available practices [Internet].

[49] Levis J, Barlaz M (2015). Composting process modeling.

